# New York State dairy farmers’ perceptions of antibiotic use and resistance: A qualitative interview study

**DOI:** 10.1371/journal.pone.0232937

**Published:** 2020-05-27

**Authors:** Michelle Wemette, Amelia Greiner Safi, Wendy Beauvais, Kristina Ceres, Michael Shapiro, Paolo Moroni, Francis L. Welcome, Renata Ivanek

**Affiliations:** 1 Department of Population Medicine and Diagnostic Sciences, College of Veterinary Medicine, Cornell University, Ithaca, New York, United States of America; 2 Department of Communication, College of Agriculture and Life Sciences, Cornell University, Ithaca, New York, United States of America; 3 Quality Milk Production Services, Animal Health Diagnostic Center, College of Veterinary Medicine, Cornell University, Ithaca, New York, United States of America; University of Minnesota, UNITED STATES

## Abstract

Antibiotic resistance is a global problem affecting both human and animal health. Ensuring the strategic and effective use of antibiotics is paramount to combatting the emergence and spread of resistance. This study explored New York State (NYS) dairy farmers’ perceptions regarding antibiotic use in dairy farming and antibiotic resistance. Dairy farmers’ perceptions were assessed through semi-structured, in-person interviews. Twenty interviews with farm owners and/or managers of 15 conventional and five USDA certified organic dairy farms with 40 to 2,300 lactating cows were conducted. Thematic analysis was used to assess, compare and contrast transcripts for farmers’ characterization of their beliefs, values, and concerns. Conventional dairy farmers had a low level of concern about the possible impacts of on-farm antibiotic resistance on human health and believed their antibiotic use was already judicious. Generally, they believed their cattle’s health would suffer if antibiotic use were further curtailed. Conventional farmers expressed frustration over the possibility of more stringent governmental, milk cooperative, buyer, or marketer requirements for antibiotic use and associated animal welfare in the future. They attributed expanding regulations in part to misinformed consumer preferences, that farmers felt were influenced by the marketing of organic dairy products. Organic dairy farmers were generally more concerned about issues related to antibiotic resistance than conventional farmers. Both conventional and organic farmers placed emphasis on disease prevention through herd health management rather than treatment. In conclusion, the conventional NYS dairy farmers in this study were skeptical of the need for and benefits of reduced antibiotic use on their dairy farms. Interventions for farmers, delivered by a trusted source such as a veterinarian, that provide training about proper antibiotic use practices and information of possible financial benefits of refining antibiotic use may hold promise.

## 1. Introduction

Globally, efforts exist to encourage antibiotic stewardship in animal agriculture due to concerns that overuse and misuse of antibiotics contribute to the development of bacterial resistance to antibiotics, including those important for treating human disease. Antibiotic use in food animals may promote the development of antibiotic-resistant bacteria that can be transmitted to humans through foodborne pathways such as contaminated animal products which are inappropriately cooked or handled [1] [2]. Other means of exposure include consumption of crops contaminated by manure fertilizers or water containing animal feces [3]. In fact, U.S. Centers for Disease Control and Prevention has classified drug-resistant non-typhoidal *Salmonella* and drug-resistant *Campylobacter* as serious health threats [3]. In addition to foodborne transmission of antibiotic-resistant bacteria, research suggests that certain resistant bacteria, such as *Staphylococcus aureus* (MRSA), can also be transmitted through direct contact with food animals [4] [5] [6].Finally, concern exists about contamination of the environment (soil and water) with effluent and manure from food animals on farms which people can then come into contact with in the environment [7].

Given the various potential routes of human exposure to antibiotic-resistant bacteria from animal agriculture, it is prudent to consider how to optimize antibiotic use in animal agriculture in order to minimize the development of antibiotic resistance. Farmers are important stakeholders in such discussions, especially since they are likely to experience any negative consequences that arise as a result of attempts to alter antibiotic use. Concerns have been raised that the push to reduce antibiotic use could have negative unintended consequences on food safety, animal health, production, and the economic viability of farming [8]. Despite the important role farmers occupy in efforts to reduce antibiotic use in animal agriculture, little understanding exists regarding farm owners’/managers’, particularly dairy farmers’, perceptions of their antibiotic use and antibiotic resistance, especially in light of recent regulations (elaborated further below).

### 1.1 Existing research on dairy farmers’ antibiotic use practices

The subject of dairy farmers’ perceptions of their antibiotic use has received even less attention than their perceptions of antibiotic resistance. One study of Wisconsin dairy farmers found that 80% believed they used the “right amount” of antibiotics [9]. This assessment is significant as previous research has suggested that there is potential for improvement in dairy farmers’ antibiotic use practices, particularly by using written treatment protocols [10] [11] [12] [13] [14]. Other areas of improvement specifically related to mastitis have also been identified. Mastitis was the disease most frequently treated with antibiotics on dairy farms from 2002 to 2007 [15]. Evidence suggests that there is room for improvement in terms of better use of diagnostics and antibiotics. A 2017 study of dairy farms in the eastern U.S., found that just 18% of surveyed dairy farmers frequently or always had bacteriological cultures performed on milk samples from cows with clinical mastitis, 49% reviewed animal records before providing mastitis treatment, 56% maintained records on cows treated for mastitis, and 70.9% treated cows with the full course of antibiotics [14].

Within dairy farming, adopting new strategies for mastitis treatment and control has the potential to reduce overall antibiotic use. Intramammary antibiotics are frequently used for both the prevention and treatment of mastitis. Related to that, pathogen-based (also known as culture-based) treatment of mastitis has garnered much interest. Using this strategy, milk from cows suspected of having mastitis is cultured and treatment decisions are made based on the results. According to a 2014 study conducted by the United States Department of Agriculture (USDA), 40.6% of US dairy operations use culture and antimicrobial sensitivity testing to guide antibiotic treatment [16]. Compared to an immediate blanket antibiotic treatment of all cows with mastitis, research suggests a delayed selective pathogen-based treatment has the potential to reduce the use of antimicrobials [17].

The use of antibiotics at dry-off (cessation of lactation) has also been considered as an area for potential antibiotic use reduction [18]. Blanket dry cow therapy (BDCT) refers to treating all cows entering their dry period with intramammary antibiotics in an effort to cure and prevent intramammary infections. However, in an effort to reduce antibiotic use, some have proposed selective dry cow therapy (SDCT) be utilized instead. Selective dry cow therapy (SDCT) involves only treating cows with mastitis, at a determined level of risk for mastitis, or meeting some other treatment criteria with intramammary antibiotics. The feasibility and impact of SDCT is subject to much discussion [18] [19]. A 2014 USDA survey study showed that 90.8% of U.S. dairy operations use intramammary antimicrobials at dry-off, and 93% of US dairy cows are treated with them [16].

### 1.2 Existing research on dairy farmers’ perceptions of antibiotic use and resistance

Despite opportunities for change, previous research with U.S. dairy farmers suggests that they are not concerned about the impact of antibiotic use or overuse on the development of antibiotic resistance in dairy cattle or humans [10] [11]. In one survey of dairy farmers, 29% agreed that antibiotic use in agriculture makes it harder to treat infections in livestock in the future and only 7% agreed that antibiotic use in livestock leads to bacterial infections in people that are difficult to treat [11]. A separate survey showed that 86% of dairy farmers were not concerned about the overuse of antibiotics in animals causing antibiotic resistance in farm workers [10]. In spite of this lack of concern, a separate study reported that farmers’ intentions to use antibiotics prudently were associated with “saving money on labor and treatment, ability to fit into daily routine, and effectiveness with veterinary guidance” [20]. Farmers’ interest in using antibiotics prudently, if they felt capable of doing so, combined with their lack of concern for resistance and belief of most that they are using antibiotics appropriately warrants further investigation.

### 1.3 Regulations on antibiotic use

Strict regulations govern dairy farmers’ use of antibiotics. Namely, legislation requires milk from cows treated with certain antibiotics to be discarded instead of sold for profit. On top of this loss of profit, there are steep penalties in the event of a violation. Milk from cows treated with some drugs must be withheld from markets for a given length of time to ensure that drug residues do not occur in milk [21]. The Pasteurized Milk Ordinance (PMO) pertains to Grade A milk and requires that industry screen all bulk milk pickup tanks and raw milk supplies for beta lactam antibiotic residues [21]. Milk testing positive for beta-lactam residues cannot be sold for human consumption [21].

As previously noted, dairy farmers’ perceptions of recently-implemented U.S. regulations regarding antibiotic use have received limited exploration, particularly in NYS. In response to concerns about the development of antibiotic resistance due to antibiotic use in animal agriculture in the U.S., the U.S. Food and Drug Administration (FDA) fully implemented changes described in their Guidance for Industry 213 (GFI 213) document on January 1, 2017 [22]. The two primary changes included (1) no longer approving the use of medically important drugs for production uses (e.g. growth promotion) and (2) establishing veterinary oversight for the use of medically important antibiotics in the feed or water of food producing animals [22]. The latter was accomplished via the Veterinary Feed Directive (VFD) final rule, which was made effective in October 2015 and fully implemented on January 1, 2017 [23]. The VFD mandates veterinary oversight for the use of medically important antibiotics administered to food animals through feed or water [23]. All use of such now requires a VFD approval [23]. These changes were based on judicious antibiotic use principles for food producing animals outlined in their earlier GFI 209 document [24]. There is very little information about dairy farmers’ perceptions of VFD. The U.S. is not alone in reconsidering the role of antibiotic use in animal agriculture. On a global scale, the WHO released guidelines in 2017 for the use of antimicrobials in food animals [25]. Recommendations made in the guidelines included restriction of the use of medically important antibiotics for prevention and control of infectious disease in food animals [25].

The objective of this study was to explore New York State (NYS) dairy farmers’ perceptions regarding antibiotic use and resistance in dairy farming, including in light of the recent implementation of the VFD. Dairy farming is a major agricultural industry in the U.S.; NYS is the third largest dairy producer in the country [26]. We specifically sought to identify opportunities, barriers and possible strategies to advance antibiotic stewardship in the context of dairy farming in the U.S. To address these goals, we used a semi-structured interviewing approach. This qualitative interview design (compared to a survey, for example) allow us the flexibility to pursue new areas of inquiry that emerge during the interview; further, it enables participants to express their views in their own terms, rather than selecting among predetermined options. To our knowledge, the perceptions of NYS dairy farmers pertaining to antibiotic use and resistance have not yet been formally explored.

## 2. Materials and methods

### 2.1 Sampling and recruitment

Purposive sampling [27] was used to recruit dairy farmer owners and/or managers, hereby referred to as dairy farmers and/or participants, in NYS. Potential study participants were identified from conventional and organic dairy farms participating in programs offered by Cornell University’s Quality Milk Production Services (QMPS) by an employee in the program and author of the study (F.L.W.). Quality Milk Production Services (QMPS) is a section of the NYS Animal Health Diagnostic Laboratory at the College of Veterinary Medicine, Cornell University. The program was initially created as the New York Mastitis Control Program and has been in existence since 1946. Resources of the program are available to all dairy producers in NYS through the central laboratory (Cornell University) or one of the three regional labs. NYS has approximately 4,500 licensed dairy farms and approximately 20% to 30% are visited by QMPS each year. The primary skills provided by QMPS to farmers and their veterinarians include diagnostic services (pathogen identification), mastitis and udder health risk assessment and education and training of farm personnel. Farmers participate in QMPS programs for a range of reasons. Some are required to do so because of milk quality issues while others are participating voluntarily because they are progressive farmers interested in improving. Study participants could have been from either of these groups.

F.L.W was asked to gather a list of dairy producers with a broad range of characteristics including farm size, management style and production philosophy (conventional, organic, grazing). F.L.W. created a list of 25 farms to contact for interviews, including farms of variable sizes and management styles to reflect the scope of dairy farms in NYS. Those identified were then recruited in one of two ways: (1) they were contacted via telephone by F.L.W. who described the study and assessed willingness to participate before providing the interested farmers’ contact information to the first author (M.W.) or (2) they were contacted via telephone directly by M.W. The first 20 farms for which contact with a person was made via telephone agreed to participate in the study. Interview dates and times were arranged by the interviewer (M.W.). Interviewees chose their preferred location of the interview, which was generally their farm and/or home.

### 2.2 Data collection

A semi-structured interview guide (S1 Appendix) was developed among authors A.G.S., M.W., R.I., W.B., K.C., P.M., M.S., and F.L.W. with the goal of better understanding participants’ perceptions of antibiotic use and resistance. The interview guide consisted of broad questions concerning participant/farm characteristics, management of health problems in dairy cattle, antibiotic use, participant’s attitudes towards changing their antibiotic use behavior, knowledge of antibiotics and antibiotic resistance, and attitudes towards regulations regarding antibiotic use. The interview guide was piloted by authors W.B. and K.C. with a director of a teaching dairy farm.

Initially, the study focused only on conventional dairy farmers and excluded organic farmers from enrollment because of restrictions on antibiotic use placed on farms with USDA organic certification. However, after 12 interviews with conventional dairy farmers, which included mention of organic practices, the research team recognized that including organic farmers would permit a more nuanced understanding of dairy farmers’ perceptions. A decision was made to enroll organic farmers along with conventional farmers. At the same time, based on iterative analysis and emerging themes, the interview guide was modified to explore in more depth specific topics identified during the first 12 interviews. The second version of the interview guide (S2 Appendix) was administered to eight farmers representing 5 organic and 3 conventional farms.

While major topic areas of the interview guides were covered during interviews, as a result of participant time constraints and the open-ended nature of the questions, not all individual questions or prompts were always addressed in each interview. Due to time and resource availability the enrollment was stopped when we reached 20 interviews, at which point saturation on many topics was also reached. Saturation was deemed to be reached when no novel themes emerged during analysis of the last few interviews.

Semi-structured interviews were conducted during June and July of 2017 and 2018 by the first author (M.W.), who is also a veterinary student enrolled at Cornell University’s College of Veterinary Medicine. They were conducted in person at participants’ farms and intended to be approximately one hour in length. Interviews lasted between 27 minutes and 89 minutes with a median of 54 minutes. Participants provided written consent at the start of the interview. The interviews were audio recorded and transcribed by the Survey Research Institute of Cornell University. The study was approved by the Cornell University Institutional Review Board for Human Participants (IRB protocol #1705007138).

### 2.3 Data analysis

Interviews were analyzed using thematic analysis [28] using QSR NVivo 11. All interview transcripts were read initially by M.W. as they were transcribed and were then also reviewed by A.G.S. and R.I. to gain familiarity with their content and to inform subsequent interviews. M.W., A.G.S., W.B., and R.I. collaborated to develop a codebook based on questions of a priori interest (i.e. perceptions of judicious use) and emergent themes (views on prevention, concerns about consumers). Initial input on the themes and codes was provided by other authors (M.S., F.L.W.) who also reviewed some of the transcripts. The prospective list of codes was discussed until agreement was reached on the final set (S3 Appendix). The topic-level codes were subsequently applied to the transcripts by M.W. in consultation with A.G.S. and further refined as necessary. This method of coding enables the participants’ exact words to be captured and organized for analysis. The software pulled all quotes affiliated with a topic (i.e. prevention); once gathered, they were analyzed by M.W. to identify higher-order themes. The team looked for similarities and differences within groups (i.e., among all conventional farmers) and between groups (comparing conventional and organic farmers’ views on both a priori topics and those that emerged). To ensure rigor and quality, the groupings, themes, and comparisons were then reviewed, discussed, refined accordingly and agreed on by A.G.S. and R.I.

Basic descriptive statistics were produced using Microsoft Excel to describe the study participant characteristics. Most participants described the size of their farms in terms of the number of lactating cows on the farm. In interviews where only information on the number of cattle over 12 months of age was obtained, that quantity was multiplied by 0.70 to obtain an estimate for the number of lactating cows. If participants gave ranges, the mean of the upper and lower numbers was taken. The value of 0.70 was obtained by determining the mean percent of cows over 12 months of age that were lactating using information from five farms that provided both their number of cattle over 12 months of age as well as their number of lactating cows. It is also supported by dairy industry information [29].

## 3. Results

As is shown in Table 1, 20 interviews were conducted, representing 20 dairy farms and involving 21 dairy farmers (two people, an owner and a manager, participated in one of the interviews). Demographic and farm characteristics for these interviews are summarized in Table 1. A quarter of enrolled farms were USDA certified organic operations, and one conventional participant was planning to transition to organic. Farms ranged in size from 40 to 2,300 lactating cattle. The majority of participants were farm owners (71.4%; n = 15/21), and the remaining were managers (28.6%; n = 6/21). There were 17 male and 4 female participants. Among conventional farmers, 60% (9/15) had a protocol for antibiotic use. From our analysis, six overarching themes emerged (Table 2), each of which is described in more detail in the following paragraphs.

**Table 1:** Demographics of dairy farmers interviewed and farm characteristics.

Variable		% (number/total)^a^
Farm-Level Characteristics (n = 20)		
Farm type	Conventional^b^	75 (15/20)
	Organic	25 (5/20)
Farm size (Number of lactating dairy cows)	<200	45 (9/20)
	200–699	25 (5/20)
	>700	30 (6/20)
Written protocol for antibiotic use among conventional farms	Yes^c^	60 (9/15)
	No	40 (6/15)
**Interviewee-Level Characteristics** (n = 21)		
Role on farm	Owner	71 (15/21)
	Manager	29 (6/21)
Gender	Male	81 (17/21)
	Female	19 (4/21)
Raised on a dairy farm	Yes	70 (14/21)
	No	30 (6/21)
	No answer	(1/21)
Plans to stay in dairy business for foreseeable future ^d^	Yes	67 (14/21)
	No-retiring	14 (3/21)
	Uncertain	5 (1/21)
	No answer	14 (3/21)

^a^ Reflects 20 conducted farm-level interviews with a total of 21 participants

^b^ One farm was transitioning to organic

^c^ One of the 9 farms had a protocol for the treatment of calves only

^d^ These variables arose in the course of the conversation or weren’t part of a primary question, and therefore the sample size was limited to the number of participants who brought the topic up.

**Table 2:** Overarching themes that emerged from the interviews with dairy farmers.

Identified Overarching Themes	
1	Among conventional farmers, concerns about antibiotic resistance do not rank highly, what is most salient is near term impacts to their livestock should antibiotics lose their efficacy
2	Conventional farmers perceive their existing use of antibiotics as meeting “judicious use” standards and that further reductions would threaten animal welfare
3	Herd health management was repeatedly emphasized as an effective preventive tool that could limit the need for antibiotics, but financial and other limitations impeded facility and other improvements
4	Organic farmers express more concern about antibiotic resistance and human health implications; both conventional and organic farmers support views on antibiotic use that are in line with their respective business practices (i.e. that organic farmers can never sell milk from a cow treated with antibiotics and conventional farmers can, after a withholding period has been met)
5	There was widespread concern about the impact of marketing and consumer perceptions on dairy; notably, many farmers believed that consumer misperceptions were driving regulation
6	Conventional farmers possess mixed views of current regulatory efforts to control antibiotic use and fear further restrictions on their antibiotic use

*Theme 1*: *Among conventional farmers, concerns about antibiotic resistance do not rank highly, what is most salient is near term impacts to their livestock should antibiotics lose their efficacy*

Farmers generally properly defined both antibiotics and antibiotic resistance. Descriptions of antibiotic resistance often incorporated explanations of how bacteria could become resistant to antibiotics or how it resulted in loss of effectiveness of the antibiotic. Two participants, however, provided explanations of antibiotic resistance that were not fully correct, describing antibiotic resistance as the animal “resisting” the antibiotic or becoming “immune” to it. When asked to elaborate their understanding of antibiotic resistance, some participants associated the term antibiotic with regulation, withholding periods, lost revenue, antibiotic resistance (an organic participant), and/or the ability to save a cow.

Conventional farmers do have concerns surrounding antibiotic use and resistance, particularly for their animals’ health if an antibiotic no longer “works”.

“Either using the same drug all the time, or using blanket treatments, I think are both pretty dangerous for introducing the antibiotic resistance. I mean, from way back—and that’s been a concern in human medicine, but then also in animals—about having less and less drugs that are available to be treatments to things.” (Interview 19, conventional)

Conventional participants expressed various levels of concern about antibiotic resistance on the farm level. Some said their level of antibiotic use was very low, so it was not a major concern for them. In regard to potential impacts of antibiotic use in dairy cattle on human health, most participants expressed little concern about possible impacts occurring either via antibiotic resistance or antibiotic residues in milk, but did note concern about on-farm impacts.

“Not with the drugs that we are using. Unless somebody told me that one of the three drugs that we use is critical for treating some humans somewhere—if I was to find that out, then I would be concerned. That’s not the case to the best of my knowledge at this point, so I’m not.” (Interview 4, conventional)

“Then the fourth [benefit of using less antibiotics] might be antibiotic resistance. In other words if you are over using it, then it’s not going to work for you anymore. Like I said, it’s more people on the human side that are concerned about that. If we are using too much medicine in our cows it’s going to somehow create a bacteria or something that’s going to affect humans and we won’t have the right drug to treat it in humans. I should be concerned, but I’m not overly concerned about that. But, I’m concerned about my herd, if you were overdoing it, then that would at some point make it ineffective.” (Interview 6, conventional)

It is unclear whether conventional participants concerned about antibiotic resistance on dairy farms thought humans could be impacted by this. One such participant was unaware that there was concern about the impacts of antibiotic resistance in agriculture negatively impacting humans. Other responses to the term antibiotic resistance included that it was “overhyped” and that it meant new antibiotics needed to be developed.

“Sometimes I think it’s [antibiotic resistance] a little bit overinflated but obviously I’m not a doctor or vet to know… What you can do but… Sometimes I think it’s overhyped.” (Interview 20, conventional)

Some mentioned concern about antibiotic resistance, but it was in the context of human healthcare, overprescribing by physicians and not connected to animal agriculture:

“I don’t know if I’d have an opinion on any resistance. It would be more likely from the human-side of things. I mean, not the animal-side of things. There’s no antibiotics in any milk, so overprescribing, I guess, would be the biggest cause of resistance in the human population.” (Interview 15, organic)

“So that’s why I said, like having people are concerned of going to the hospital, the doctor came in and said, “It’s a strain that’s hard to treat.” I would say, “Oh no, I’ve read about these.” Or, “No, I knew somebody that had it.” But as far as on the farm, I don’t hear just a lot. I guess it’s a conversation the vet and I haven’t really had. I feel I use that little antibiotics on the farm that I don’t have that much of a fear of antibiotic resistant disease.” (Interview 18, conventional)

Finally, when asked how antibiotic resistance ranked among other operational concerns, farmers responded they were generally addressing more immediate concerns.

“Low [laughs]. We are mostly worried about keeping the tractors going.” (Interview 2, conventional)

Antibiotics were a major source of concern when it came to residues getting into the milk supply. In response to a question how antibiotic use ranks relative to other concerns about managing a dairy farm, one farmer answered this way with “it” being about residues.

“It’s probably the biggest concern I have as far as what could go wrong in a day. Selling a treated cow, or putting a treated cow in the milk supply. It’s pretty well-managed so I don’t lose sleep over it. But it is probably the top concern I have as far as what could go wrong during a day, besides the cows getting out.” (Interview 3, conventional)

*Theme 2*: *Conventional farmers perceive their existing use of antibiotics as meeting “judicious use” standards and that further reductions would threaten animal welfare*

Most conventional participants interpreted judicious antibiotic use to mean using antibiotics only when needed, not more or less frequently. The following quote is representative of the feedback we heard:

“Think. Think before you use it. I mean, only use it if it’s needed. That’s what judicious use means. If you don’t need it, don’t use it. If you don’t know why you’re treating it, or what you’re treating it for, don’t use it.” (Interview 5, conventional transitioning to organic)

Some participants provided greater detail and specified that judicious use of antibiotics included avoiding blanket use and following directions regarding use. Blanket use of an antibiotic in livestock generally refers to administering an antibiotic to a group of animals for disease prevention or treatment, regardless of whether a specific individual is suspected to have a given disease. Other farmers’ descriptions incorporated prevention of disease, use of diagnostic tests, observing treatment effectiveness, using the least amount, and disease prevention. One participant associated the “judicious” with the consequences that they could face resulting from shipping a load of milk with antibiotic residues.

Conventional farmers perceived their antibiotic use on dairy farms to be generally minimal, optimal, or improving. Several argued that further decreasing their antibiotic use would compromise animal welfare and/or cause them to cull more cattle.

“You have to treat her. It’s no different than would you no give your kids antibiotics if they were sick? It’s not like we go around giving our kids antibiotics for no reason. Same with the cows, we don’t give them antibiotics for no reason. If they are sick they need to be treated.” (Interview 2, conventional)

Overall, participants indicated that they used antibiotics only when necessary, their level of use was low/minimal, and/or that they were considering using less. Some emphasized that they, as well as dairy farmers in general, prefer not to use antibiotics because of the many drawbacks of antibiotic use. Examples of drawbacks included the cost of the antibiotic itself, lost milk yield due to having to potentially discard milk from treated cows, and time spent managing treated cattle to prevent them or their products from entering the food supply.

“Yeah. I try to tell people, when I get the opportunity, that I think the misconception that is out there is that we use antibiotics very frequently, and without merit, and that sort of thing. And I try to make them aware that we try to keep a very healthy animal so we don’t have to use antibiotics. Because antibiotics cost us money in lost product …” (Interview 18, conventional)

When asked about perceptions of other farmers’ antibiotic use practices, most participants stated either that they did not know about other’s practices or stated that dairy farm owners and/or managers prefer to use as little antibiotics as possible. While most dairy farmers believed that however, some participants, including those who reported that most dairy farmers preferred to not use antibiotics if they were not needed, stated that they knew of other farmers whose antibiotic use practices were not optimal due to overuse or failure to properly follow withhold times or directions on drug labels. In fact, one of this study’s participants suggested they administered a greater dose of antibiotic than recommended by the directions on the label if they felt they were treating a more serious illness because they believed it would help the animal recover faster.

*Theme 3*: *Herd health management was repeatedly emphasized as an effective preventive tool that could limit the need for antibiotics, but financial and other limitations impeded facility and other improvements*

Both organic and conventional farmers emphasized the importance of disease prevention and maintenance of herd health as opposed to treatment and believed that focusing on prevention lessened the need for antibiotic treatment.

“I think the main impetus, and [the herd manager] certainly does this beautifully, is to keep the cows at a high plane of health so you don’t have to use antibiotics. That includes doing preventive inoculations, a clean place for them sleep, and excellent feed.” (Interview 2, conventional)

“I’m trying to find ways to use less [antibiotics]. I know other managers would like to use fewer antibiotics. The only way you are really going to do that is just through management, is through better prevention.” (Interview 3, conventional)

They described management or facility changes they hoped to make. Most changes were related to improvements in infrastructure and facilities, often with a focus on calf and dry cow facilities. Management changes included improved biosecurity, reducing overcrowding, and finding management strategies to prevent pneumonia in calves (better colostrum delivery, having a colostrum pasteurization system). For many farms, pathogen-based mastitis therapy was an important part of managing mastitis in cows. All farmers who spoke of this treatment strategy spoke positively of it, and some mentioned it had allowed them to operate more efficiently and reduce their antibiotic use.

“Yeah, it’s that blanket treating everybody that didn’t do a damn thing, not in reality. Better off following [pathogen based mastitis therapy]—All the research and all the data has shown, this bacteria can be treated, this can’t be, and this is how you handle it. Use a lot less drugs and a lot less time out on the table too, cow individually.” (Interview 20, conventional)

Some farmers did mention that other farmers may face logistical barriers (transport of sample) or time/labor barriers to participation in the program.

Facility changes included improving barn ventilation (particularly for calf facilities), building a new barn at a more convenient location, building a dry cow barn or improving an existing dry cow barn, having calving pens, having better calf facilities in general, transitioning to sand bedding for cows, having greater available acreage for cattle on pasture, and general facility renovations/upgrades. Some were also exploring new tools, such as rumination collars, they hoped to utilize in order to improve the health of their cattle. They expressed the sentiment that with greater resources, especially related to infrastructure/facilities, they could further improve the health of their cattle. Many also mentioned that there were a variety of ways in which farmers might improve herd health through management changes, but that those changes required commitment and tradeoffs.

“If your focus, is, ‘We want to use less antibiotics,’ then there’s a hundred different ways to do it. Where it may cost more in labor, or management practices, or something like that. But if that’s the focus, I think any place, including us, can probably find different ways to do it to be able to reduce.” (Interview 19, conventional)

*Theme 4*: *Organic farmers express more concern about antibiotic resistance and human health implications; both conventional and organic farmers support views on antibiotic use that are in line with their respective business practices (i.e. that organic farmers can never sell milk from a cow treated with antibiotics and conventional farmers can, after a withholding period has been met)*

Regulations for antibiotic use in organic farming differ starkly from those for conventional farming, namely that once treated with antibiotics, a cow’s milk can never be sold as organic again. Treatment, however, cannot be withheld from cattle requiring it. Organic participants' descriptions of judicious antibiotic use were more detailed than those of conventional participants and included that antibiotics should not be used to increase production (growth promotor or milk production enhancer), used for prevention, used as a blanket treatment, or fed in the form of medicated feeds.

“I think only for therapy, for known extant medical issues. Not a routine blanket kind of use, just to prevent something that may never be a problem. And/or it should not be used as a growth promotant, or to increase production—either growth or milk production.” (Interview 13, organic)

Others commented on concerns about antibiotic residues entering consumer products, despite regulations, practices and penalties designed to prevent this–but felt that pressure to be “pro all milk, not just organic milk” made this difficult to communicate (Interview 14, organic). One organic participant responded differently to this inquiry about judicious antibiotic use by raising the concern that on organic farms antibiotic treatment may be delayed too long to maximize profit since animals treated with antibiotics lose value as they need to be sold to the non-organic market. In general, delayed treatment can result in poorer clinical outcomes and/or prolonged animal suffering.

When discussing antibiotic resistance, in contrast to conventional participants, organic farmers had higher levels of concern about antibiotic resistance. They also often framed antibiotic resistance as a larger environmental and public health issue (e.g., consistent with [7]), rather than focusing on farm-based complications issues.

“But I’m concerned for the health—for the human health side. That we don’t lose—that we as a people don’t lose effective use of antibiotics that are important to human treatment. As well as just all those antibiotics getting in the waterways. I mean not only from animals, but from all the pharmaceuticals from people too. But I mean, certainly if animals are routinely being treated, you know, it’s got to end up in the waterway sometime. Because I don’t think the body’s metabolizing it all or breaking it all down.” (Interview 13, organic)

There was some similar doubt amongst organic farmers about the extent to which dairy farming was contributing to antibiotic resistance, particularly the human health impacts. One organic participant said that human health impacts of antibiotic resistance were primarily due to overprescribing in human medicine, not overuse in dairy farming.

*Theme 5*: *There was widespread concern about the impact of marketing and consumer perceptions on dairy; notably, many farmers believed that consumer misperceptions were driving regulation*

All conventional farmers expressed concern about consumers’ lack of knowledge and/or misconceptions about the dairy industry, especially in regard to cattle welfare, and the impact of this misperception about antibiotics on both purchasing and regulation.

“I think they [consumers] are under the impression that their milk comes from factory farms where the cow’s welfare isn’t taken into account. I’ve got relatives … that are just amazed at how well we treat the cows when they come see the farm. But before they were here, they were under a totally different impression. We got to make the public aware of what we do.” (Interview 3, conventional)

“I think it’s regulations and definitely something that’s consumer-driven—an awareness of consumers and what… the fact of the matter is that with consumers, perception is reality.” (Interview 5, conventional transitioning to organic)

“Bottom line, the consumer is what drives everything and that’s why we do things the way we do. But because of uneducated people it makes it harder for us to do our job the way we should be able to do it.” (Interview 2, conventional)

Conventional farmers commented that antibiotic overuse is not as commonplace as consumers believe and that not using antibiotics when needed is inhumane and a welfare concern.

“Well I still think that a lot of people think you can give a cow antibiotics and ship the milk. Obviously we can’t do that. So that’s something consumers should realize that… And I think it’s a good change what we’re going to, trying to minimize the amount of antibiotics we’re using. But in the same token, you know if your child has an ear infection and you know what antibiotic can cure it, you’re going to give it to them. You’re not going to say, “Oh, it’s antibiotic. No, stay away.” So I think in the right circumstances I mean it promotes health. Just don’t abuse it.” (Interview 20, conventional)

Further, they expressed concern about consumer worries about residues in the milk and emphasized the safety mechanisms that are in place to prevent consumer exposure to antibiotic residues in milk because of the withholding periods, testing, and high penalties for violation.

“So, being that I grew up around animals and food operations, then I probably don’t have a lot of the misconceptions other people do. Like if a label says that it’s antibiotic free, it’s like, “Well of course it’s antibiotic free. Any milk you get’s going to be antibiotic free.” So, that’s sort of a misnomer. But, because it seems so natural to me like, that’s how it’s going to be, then I can’t understand how anybody would ever think any different.” (Interview 19, conventional)

“Some of them should know that every drop of milk that goes to a processing plant or a bottling plant is tested for antibiotics. There’s none in it. I think that’s the biggest misconception that consumers have, is that if they are not buying an organic product, then what they are buying has been laced with antibiotics in some form or other. They should know that that’s not the case. It’s all tested and that there’s nothing there. That is what I would like people to know.” (Interview 4, conventional)

Indeed, previous studies summarizing and investigating the presence of antibiotic residues in milk have found occurrence of residues to be low [30] [31]. However, this concern mirrors some of the publics’ reasons for purchasing organic and perceptions of it described previously [32] [33]. These views are perhaps not surprising given the price premium that products raised without antibiotics currently enjoy and such views reinforce their current business model. Some farmers suggested that both consumer concerns and misconceptions regarding the dairy industry–from antibiotics to animal welfare- had driven increasing regulation and requirements, especially those established by milk cooperatives, milk buyers, and marketers.

As previous quotes have hinted, conventional farmers are concerned about organic farming practices and the impact on consumer perceptions of organic dairy product marketing. Some conventional participants expressed disagreement with organic farming rules and requirements regarding antibiotic use. These participants are concerned that organic farming requirements create a financial incentive to not treat sick animals or to wait longer to treat, potentially compromising animal welfare.

Conventional participants also mentioned that the organic designation is simply a marketing tool and meaningless in regard to milk quality.

“I’m sorry, I’m totally against organic, so I think that’s just a whole foo-foo thing, because you’re just paying for… I mean, our ancestors lived hundreds of years and they didn’t have organic. I think it’s just a marketing push…” (Interview 11, conventional)

“… If I was talking to a consumer I’d say—if you go back to organics—I would say, “Why do you want to buy milk taken from a cow that’s sick? What is your reason for wanting this? Why do you want to spend another dollar or whatever for milk to drink it from a sick cow?” And I still don’t understand why people want to have it but apparently there’s demand for it. ….” (Interview 8, conventional)

Finally, several farmers expressed suspicion about whether organic farmers were fully following organic requirements. They suggested that organic farmers were actually using antibiotics in violation of organic requirements–treating sick cows and selling that milk as organic.

*Theme 6*: *Conventional farmers possess mixed views of current regulatory efforts to control antibiotic use and fear further restrictions on their antibiotic use*

Participants reported that in general, government regulations (including the VFD) and rules established by milk cooperatives, buyers, and marketers have been workable and not overly burdensome to implement.

“I don’t have a big problem with that [the VFD]. If you’re going to be using antibiotics, you should… your vet should be involved.” (Interview 5, conventional transitioning to organic)

“Certainly, it makes us more—I think it just makes us more conscious of the use. So… I haven’t seen where that’s—I can’t see where that’s a bad thing. I mean, I guess a greater concern was if that sort of creep kept happening, where there are more and more restrictions. Which I guess is hard to say when it’s been too much. But it doesn’t really feel like it’s been stuff that’s been burdensome.” (Interview 19, conventional)

“Oh… Well… I’d say I don’t have a great opinion on it, other than it’s kind of cumbersome. . . . it’s doing what it intended and it’s limiting the amount of antibiotics in feed that you can use. So if that’s the goal, then it certainly is, I’m sure, working, because it’s more of a hassle now to get a script and write down the exact days and how much you’re doing it. So I think most farms have pretty much gotten away from it. Probably.” (Interview 20, conventional)

Overall, farmers had varying opinions of the VFD and its purpose; some viewed it as a promoting positive change while others thought it was unnecessary and just an additional requirement to be met that came with costs.

“I don’t know negative or positive because it could make some farms do a better job. But it would make other farms that are doing a good job have to hire an extra person or two to fill in the gaps. I’ll spend two hours a week writing SOP’s and making sure people are up to date on what the guidelines say they are supposed to be doing. Where five years ago, I would just go out and talk to everybody and I wouldn’t have had to write everything down. I think in the future I’m just going to have to do more paperwork, which is going to take me away from being with the cows. So I’m going to have to hire a new half-a-person or a person that I wouldn’t have had to. It will be less efficient.” (Interview 3, conventional)

Some participants disliked rules and regulations because they viewed them as interference into their work and business.

“I guess for me, I’ve gone from being just a farmer and doing the work to doing more labor management. … And environmental and other government—I call it intrusion—into our business. People say all these rules are good, but if you could keep the banker happy and the milk inspector happy when I started—that’s all you had to worry about. I didn’t have hired labor, so you didn’t have to keep your hired men happy, you didn’t have to keep the DEC [NYS Department of Environmental Conservation] happy, you didn’t have to keep the FDA happy… All these rules that are being imposed on us now, and public pressure. We try to do as good a job as we can, but all the scrutiny is kind of a challenge for me.” (Interview 6, conventional)

This farmer notes the conflict about the potential value of additional advice, but the potential loss of autonomy and the possibility for further restrictions on antibiotic use:

“Yeah. So, I think I’m divided on it. Part of me thinks, again, having that extra conversation with a veterinarian that is prescribing these drugs is really important, because he needs to be conscious of how we’re using it, and that we’re not being irresponsible, using his name to use drugs that shouldn’t be used that way. So having his opinion one more time of, “Alright, well these calves should be treated,” or “these calves shouldn’t be treated,” is really good. But then, the libertarian in me says, “Hey, you know, how much more should people infringe on the way that we—the practices that we think are going to be the best for the calves.” So I’m just sort of evenly divided on the benefits of it, but then also that little of bit of a creep in of… Somebody coming in—between the relationship of us and our vet, that pushes us and wants to make those type of things more restricted. I can’t say that that’s a good thing or a bad thing, but I think it’s just a concern.” (Interview 19, conventional)

As alluded to in the last part of the above quote, a frequently mentioned concern was the possibility of further limitations on which antibiotics could be used or how much, and how much more difficult further restrictions would make their work without providing visible benefits.

“I will tell you this, we don’t have very good luck treating pneumonia anymore. I think that’s because they’ve taken all the good antibiotics away from us. That’s one area that if we do get a developed cow with pneumonia, we just don’t have the arsenal of drugs in my mind to treat her the way she needs to be treated.” (Interview 8, conventional)

“I understand why they’re doing it, and I don’t disagree with it. It’s just… Some of it is, I guess the hardest part is, I understand why they’re doing it all, with antibiotics, but it’s getting down to the point where, pretty soon we’re not going to have a hell of a lot of choices. I just don’t it want it to hurt the animal…” (Interview 20, conventional)

## Discussion

Conventional dairy farmers’ general perceptions of antibiotic resistance expressed during these interviews reflect those described in previous literature, though our findings include stratification of farms by conventional versus organic status. Most conventional farmers in our study did not express concern about and/or belief in potential consequences of antibiotic use in dairy farming on human health, a finding consistent with other research on the topic [10] [11] [34]. Though concerns over the impact of antibiotic use on human health were raised more frequently among organic farmers, such concerns align strongly with their business model. For conventional farmers, the threat of antibiotic resistance did register in more localized ways, however, via concern about the development of on-farm antibiotic resistance in dairy cattle, a finding similar to other work [11] [34].

One defining feature of this study is that it also explored participants’ personal definitions of judicious antibiotic use and included questions that prompted them to evaluate their own use. Perhaps not unexpectedly, participants’ personal definitions of judicious antibiotic use focused on minimizing antibiotic use. This is similar to the definition provided in the Guidance for Industry #209 which defines judicious use as refraining from “unnecessary or inappropriate use” [24].

While the definitions offered by farmers and the FDA are similar, they are not the same. Minimizing antibiotic use may in fact be *injudicious*, if antibiotic treatment is not provided when needed or in insufficient quantities or duration to address the concern. Conventional dairy farmers perceived their use of antibiotics as judicious, a perception seen in other studies as well [35]. However, these perceptions may not be entirely consistent with their behavior. During the course of the interview, a few participants described antibiotic use practices that did not align with judicious use principles; others described situations in which it was unclear if antibiotic use was warranted or the best option for treatment of disease. These revelations coupled with the confusion about antibiotic resistance among some participants, suggests a possible role for further discussion and training regarding antibiotic resistance and good antibiotic use practices, as well as identification of specific reasons for the non-judicious use.

Conventional farmers were consistently concerned about negative impacts on cattle health and welfare if their use of antibiotics was to be further reduced particularly since they believed they already used them judiciously. Many indicated that they had a moral and/or ethical responsibility to treat cattle. Research with other populations echoes the interviewees’ sentiments and suggests most feedlot operators feel a moral obligation to their cattle to treat them with antimicrobials [36]. The way reductions or restrictions in antibiotic use might impact cattle health and welfare is difficult to determine. A systemic review of unintended consequences of antibiotic use restrictions in food producing animals found that reported effects on animal health were inconsistent between studies [8]. Further investigation into this area is needed.

Based on our findings, most conventional farmers believe they have met the goal of judicious use and are often skeptical of implications for human health. Prior research indicates that recognizing a problem and perceived responsibility to address that problem is a determinant of adoption of change [37]. Therefore, efforts to motivate further reductions in antibiotic use by appealing solely to human health concerns may fail to gain traction, as farmers do not see their use of antibiotics to be a threat. The current absence of definitive evidence that antibiotic use in dairy farming impacts human health provides a practical and communication obstacle [38]. However, participants had many other non-human health related concerns that made them interested in minimizing their antibiotic use. The primary concern was financial losses due to antibiotics use e.g., the costs of antibiotics and loss of milk during the withholding period. Therefore, if there were evidence that there were financial gains to be made via optimizing antibiotic use, these arguments would likely be more persuasive in terms of getting farmers to consider a change and be open to further conversation.

Education regarding antibiotic resistance could also potentially alter levels of concern surrounding human health impacts among a few farmers. An area that could benefit from future research might relate to farmers’ understanding of antibiotic resistance mechanisms. One participant quoted earlier was able to define both of the terms antibiotic and antibiotic resistance. However, they expressed lack of concern about antibiotic resistance because they did not believe any of the drugs they used were utilized for humans. An explanation of how bacteria can develop resistance to classes of antibiotics as opposed to a specific antibiotic might be beneficial in this case.

It may be possible to optimize antibiotic use through education leading to improved decision-making about antibiotic use. Educational efforts should underscore the financial benefits of making good antibiotic use decisions and be attuned to farmers’ commitment to maintaining cattle health and welfare. Another important way to achieve this objective is reducing disease incidence via better farm management. Many farmers expressed great interest in disease prevention, however, they believed they faced financial and other limitations to making improvements, particularly to their facilities. Farmers have been shown to be more likely to adopt changes that they perceive as feasible, effective, and that they have the knowledge to implement [37]. In addition, recognizing other constraints to participation in such programs, such as available time, is also important in any intervention design and delivery [39].

Mastitis accounts for a great deal of antibiotic use in the dairy industry [15]. Research has shown that on large farms employee management strategies (ensuring stringent compliance with milk quality protocols, giving employees a financial or other penalty for increased somatic counts, and reducing farm labor costs) are associated with lower bulk tank somatic cell counts and greater mastitis control [40]. While that study focused on mastitis, improved employee management may also be applicable to enhancing other areas of herd health management. In our study, farmers generally had a positive impression of pathogen-based mastitis therapy and believed it to be valuable, though some barriers to its uptake were identified. More research on barriers to its uptake as well as its impacts on antibiotic usage and the farm economics would be valuable.

Organic farmers seemed generally more concerned about potential negative human health impacts stemming from antibiotic use in agriculture than conventional farmers. This finding is supported by previous studies comparing organic and conventional farmers’ attitudes regarding antibiotic use [11] [34]. Organic farmers have also been shown to be less likely to agree that increasing restrictions and regulation of antibiotic use is a concern for those in the agriculture [11]. The USDA restricts antibiotic use on farms with organic certification. Products from dairy cattle administered antibiotics cannot be sold or marketed as organic [41]. However, organic farmers are not permitted to withhold antibiotic or other treatment from a cow that needs such treatment [42]. Therefore, while an organic dairy farmer may treat their cattle with antibiotics if needed, these cattle and their products cannot be sold as organic. Such cows are removed from the organic operation and may be sold for processing as non-organic meat or be sold to a conventional farm. In contrast, European Union Organic Regulations permit cattle treated with antibiotics to retain their organic status if specific restrictions related course of treatment and withdrawal periods are followed [43].

The interviews exposed tension between some conventional and organic farmers with regards to the use of antibiotics. Both sides believed their farming methods (conventional or organic) were optimal and that they were ‘good farmers’. However, some conventional farmers expressed moral concern that reducing antibiotic use could potentially threaten cattle welfare and that welfare may be compromised on some organic farms if antibiotic treatment is delayed to maintain an animal’s organic status. In contrast, some organic farmers expressed concern about human health impacts of antibiotic use in cattle, drug residues in milk, and the environment, as well as that on some conventional farms antibiotics are used to compensate for not applying methods to prevent disease in the first place. These findings are not surprising given the literature about motivations for ‘good farming’ that differ between conventional and organic farmers. Conventional farmers seem to be motivated by proficiency and timelines [44] while for organic farmers the attitudes towards environment and health play a major role [45]. Despite the motivation, both organic and conventional farmers have an underlying emphasis on the importance of maintaining a financially viable farm [44] that governs their business models.

The interviews highlighted the many stakeholders that dairy farmers must interact with and navigate among in the dairy industry. These include consumers, the government, and milk buyers. Farmers felt consumers were misinformed about various aspects of dairy farming, including antibiotic use. Consumers may indeed lack knowledge about dairy farming; in 2016, only 1.5% of the U.S. population was employed in the agriculture, forestry, fishing, and hunting sector [46]. Furthermore, conventional farmers felt that these misconceptions promoted the implementation of rules, requirements, and regulations by milk cooperative, milk buyers, and the government. Some conventional farmers expressed concern over what they felt was an increase in rules and regulations for antibiotic use. A survey study of dairy farmers found similar concerns, with 75% of conventional dairy farmer respondents indicating that they agreed that increasing restrictions and regulations for antibiotic use is a major threat for the agricultural industry [11]. Interestingly, Canadian dairy farmers have expressed similar concerns, specifically about increased government involvement in on-farm food safety [34]. Such concerns may stem from mistrust of the government and farmers’ recognition that public and governmental expectations regarding antibiotic use do not align with their own as described in existing literature [36]. A 2019 study specifically focused on evaluating dairy farmers’ perceptions of the Farmers Assuring Responsible Management Animal Care Program (FARM-ACP) [47]. FARM-ACP sets animal care program standards for dairy farmers that are intended to enhance consumer confidence in dairy programs, and over 98% of the U.S. milk supply comes from farms participating in the program [48]. One theme identified was farmers’ distrust of the program [47]. The paper suggests that this distrust may be due in part to farmers’ perceived lack of input in the program [47]. In the same study, 83.3% of participants indicated future revisions of the program needed increased input from farmers [47]. To maximize the chance for a successful intervention, substantial efforts should be made to incorporate dairy farmers in discussions of antibiotic use and resistance occurring in academia and government, especially if such discussions may have regulatory implications. This involvement would allow them to offer invaluable feedback on the practicality and consequences of solutions proposed, share their own perspectives on issues, and hopefully build trust between dairy farmers and outside stakeholders.

This study provided new information about NYS dairy farmers’ perceptions of the new VFD. Participants expressed that it takes their additional time and effort to comply with the VFD rule. Some recognized its role in promoting more judicious antibiotic use. For example, it dissuaded some participants who were using medicated feed or milk replacer in their youngstock prior to the regulation to stop using it. Others thought the new rule was unjustified and taxing. These findings overlap with those from few existing studies about the VFD in the U.S. In Tennessee, dairy farmers perceived the VFD as unnecessary and burdensome, to have affected small producers, and introduced additional costs [49]. Another study found that Tennessee dairy producers believed that the VFD had driven increased antimicrobial use, especially the use of injectable antibiotics to treat disease that had previously been prevented through the use of antimicrobials in feed [35]. Tennessee beef producers expressed similar views and concerns [50].

To help advance changes in antibiotic use, research indicates that dairy and other farmers generally prefer to receive antibiotic-related information from their veterinarians who they view as a trusted source of information [10] [35] [50] as well as food safety information [34]. This is a promising, and seemingly accessible, avenue for intervention as in our study most conventional participants did not report receiving information on antibiotic resistance from their veterinarian, and some reported receiving no information about it at all. In a previous study on the topic, only 35% of dairy farmers reported haven spoken with their veterinarians about antibiotic use in the past 6 months [10]. Lack of communication about antibiotic resistance and good antibiotic use practices between veterinarians and conventional dairy farmers may be due to veterinarians’ perceptions that farmers not being interested or receptive to that information. This potentially parallels with the relationship between physicians and patients in human medicine in which perceived pressure from patients’ parents was a barrier to pediatricians using antibiotics judiciously [51]. Given the important role of veterinarians in dairy farming, it would be valuable to better describe their opinions on antibiotic resistance, stewardship efforts, and the practicality of farmers making changes that might allow them to optimize their antibiotic use. In addition to veterinarians, cooperative extension may also be a good venue for educational interventions targeting farmers. Youth organizations such as 4-H and Future Farmers of America could also explore where information pertaining to antibiotic use might be incorporated. While education should focus on disseminating information on antibiotic resistance and good antibiotic use practices, it would also be expedient to focus on ways in which reducing antibiotic use can increase efficiency and reduce farmers’ costs as that was one of the downsides mentioned by farmers to using them. Emphasis could be placed on strategies necessary to ensuring farmers’ economic viability with judicious antibiotic use featured as a core strategy.

Complicating the relationship between conventional dairy farmers and stakeholders is the belief that the marketing of organic dairy products contributes to and reinforces consumers’ misconceptions about the dairy industry: specifically, that organic dairy farming is somehow “better”. While research does suggest that consumers may associate organic food with being healthier, free from antibiotics, hormones, and chemicals, and improved animal welfare [32] [33], it has also indicated that consumers are skeptical of product labels such as “all natural” [32]. Farmers’ concern about how consumers view their practices, however, is not unfounded: in one survey of the general public, over half of respondents indicated that they were concerned about dairy cattle welfare and generally tended to agree that there is a trade-off between profits and animal welfare in dairy farming [52]. The general public’s beliefs about organic products along with their skepticism surrounding labelling highlights a possible area for outreach to consumers that provides clarification on labelling terms (e.g. “Organic”, “Natural” and “No antibiotics”) and the opportunity for consumers to learn about modern dairy farming practices on both conventional and organic operations, and so become better informed buyers.

It is important to consider the results presented here in the light of the intended scope of the study and limitations. The purposive convenience sample of farmers from NYS was not able or intended to be representative of all NYS dairy farms, much less all dairy farmers in the U.S. Study participants were selected from farmers participating in programs offered by Cornell’s QMPS, as this is how we had access to dairy farmers. This could have potentially introduced a selection bias into our study if farmers with particular characteristics had disproportionate representation or were not representative of the general population of NYS dairy farmers. There was no way for us to compare study participants in QMPS with non-participants, and there are issues therefore that may not have emerged. The study involved a relatively small sample size, especially of organic farmers. While we do not believe this compromised the richness of our data as we reached saturation, this study design did not intend to enable an exploration of potential differences between subgroups in our sample, such as large versus small farms within each category (conventional and organic). The interview setting utilized in this study makes social desirability bias possible, due to the controversial nature of the conversation surrounding antibiotic resistance and appropriate use of antibiotics. While the veterinary student interviewer likely exerted some level of influence to report proper antibiotic use among dairy farmers, the participants may have been more forthcoming than had a veterinarian or dairy expert conducted the interviews. Our semi-structured questions did not explicitly distinguish between intramammary versus systemic antibiotic use, and therefore no differentiation could be made between antibiotics used for the treatment of mastitis versus systemic illnesses. Similarly, we did not explicitly question participants about the use of antibiotics for mastitis treatments and farmers’ opinions regarding current and emerging usage practices (pathogen-based mastitis treatment, SDCT versus BDCT). Our findings are based on farmers’ perceptions and self-report of practices. The study was not designed to observe practices and animals in order to compare those observations to what was reported in interviews about behaviors around judicious use or animal welfare. The study findings are specific to NYS, so not all themes may be relevant to dairy operations in other areas of the US.

## Conclusion

To our knowledge, this study is unique in that it formally explored the NYS dairy farmers’ perceptions of antibiotic use in dairy cattle and antibiotic resistance. The results suggest perceptions held by farmers that may be targeted for additional research and educational interventions and suggest avenues through which educational communication might occur. Given farmers’ interest in disease prevention, they would likely be amenable to interventions focused on improving the efficiency and financial viability of their operation through improved herd health practices, including optimal antibiotic use/best practices. Such interventions would likely be best delivered by a veterinarian given farmers’ trust of them. Since veterinarians have an important role to play in promoting judicious antibiotic use practices, it is vital to acquire an understanding of their own perceptions of antibiotic resistance and their interest in promoting interventions among dairy farmers.

Finally, having a way in which to quantify antibiotic use in order to better understand how judicious one’s use actually is would be valuable in an effort to promote judicious antibiotic use, as would establishing a consensus on what best practices are, given the variation. Adopting an inclusive and multidimensional approach to tackling antibiotic resistance that encourages interactions between veterinarians, medical professionals, public health professions, and farmers will help to ensure that proposed solutions are practical and amenable to all.

## Supporting information

S1 Appendix Initial guide for semi-structured interviews. (DOCX)

S2 Appendix Revised guide for semi-structured interviews. (DOCX)

S3 Appendix Codes for Interviews with dairy farmers. (DOCX)
