# Supplementary material for: New York State dairy farmers’ perceptions of antibiotic use and resistance: A qualitative interview study
Source: PLoS One. 2020 May 27;15(5):e0232937. doi: 10.1371/journal.pone.0232937 (PMC7252592; doi:10.1371/journal.pone.0232937)
Supplement: S1 Appendix — (DOCX) [file pone.0232937.s001.docx]

**Appendix 1. Initial guide for semi-structured interviews**

1. **Introductory Questions**
   1. How did you get started in farming?
   2. What’s been the biggest change you’ve experienced in the past several years in regard to operating your farm?
   3. How many *adult dairy cows (over 12 months)* are currently on your farm (including all sites)?
   4. How many years since you started working with cattle?
   5. Do you plan on continuing to work with cattle for the foreseeable future?
2. **Health problems on the farm**
   1. Who do you talk with about finding solutions to health problems on your farm?
   2. What health problems in your cattle are you most concerned about?
   3. How do you manage those problems? [prompt if necessary: vaccines, diagnostics, nutrition, environmental]
   4. Who makes decisions about health problems on the farm?
   5. In what circumstances do you speak to your veterinarian?
3. **Use of antibiotics**
   1. What do you understand the term “antibiotic” to mean?
   2. What factors do you consider when deciding whether to use antibiotics and in choosing an antibiotic (both for prevention, treatment or any other purpose)?
   3. Where do you get information about how to make these decisions?
   4. Do you have a written protocol for antibiotic use?
      1. If so, in what situations do you deviate from the protocol?
      2. If not, how do you determine how to use antibiotics?
   5. Do you have questions, points of confusion or concerns about using them?
   6. Who do you go to with questions about their use?
   7. What source is most helpful in learning about antibiotic use?
   8. Do you and your veterinarian talk about antibiotic use?
4. **Attitudes toward changing behavior**
   1. Relative to other concerns you have about the operation of your business and your responsibilities, how important is antibiotic use?
   2. Do you think dairy farmers that you know are considering using more or less antibiotics in general (both for prevention, treatment or any other purpose)?
   3. Is antibiotic use/antibiotic resistance something you talk about with others you know in the business?
   4. Assuming a dairy farmer was interested in reducing antibiotic use on their farm, what would convince them to use even less antibiotics? (evidence or motivation)
   5. Have you heard of success stories? Of failures?
      1. If so, from where?
   6. Have you considered using more or less antibiotics in the past? What did you do?
      1. What do you think the benefits would be of using even less?
      2. What are the costs or downsides of using even less?
   7. What would you want to know about other farmer’s use of antibiotics?
   8. Do you have ideas about the use of antibiotics you think others should hear?
   9. What have you considered doing or done to reduce the risk of disease? Were there things you considered but decided not to do? Why? (e.g. vaccination, quarantine, purchasing animals from known sources etc….)
   10. Have you considered management changes to decrease the use of antibiotics?
   11. Assuming you were interested in reducing antibiotic use on your farm, what would convince you to use even less antibiotics on your farm? (evidence or motivation)
5. **Knowledge/beliefs about antibiotic resistance**
   1. What does antibiotic resistance mean to you?
   2. Are you concerned about anything related to antibiotic resistance in your farm?
   3. Do you know anyone who has been affected by this (human health or animal health)?
   4. Where do you get your information about antibiotic resistance?
6. **Knowledge**
   1. How do you keep up to date or get information about current guidelines and laws (ex. VFD, other FDA laws or guidelines), especially around antibiotic use?
   2. Are FDA guidelines something you follow?
   3. What does judicious use of antibiotics mean to you?
   4. Part of the FDA’s goal to reduce antibiotic resistance is “limiting medically important antibiotic drugs to uses in food-producing animals that are considered necessary for assuring animal health” FDA guidance document #209
      1. Are you aware of the antibiotics that are considered medically important?
   5. These can be found in FDA guidance document #152 Appendix A
      1. What circumstances would lead you to use even less of the antibiotics considered medically important?
      2. Knowledge about current guidelines
   6. Are you aware of the Veterinary Feed Directive (VFD)?
      1. “A VFD drug is intended for use in animal feeds, and such use of the VFD drug is permitted only under the professional supervision of a licensed veterinarian.” Fda.gov
      2. The VFD went to effect on January 1, 2017.
   7. What do you think about this directive?
   8. How does the VFD affect the way you operate your farm?
   9. How do you get information about the VFD and other new guidelines?
7. **Concluding questions**
   1. What do you think the future of farming looks like?
   2. Do you think that antibiotic use in farms is changing, or will change, in future? How? Why?
   3. Is there anything else you’d like to talk about?
