# Supplementary material for: New York State dairy farmers’ perceptions of antibiotic use and resistance: A qualitative interview study
Source: PLoS One. 2020 May 27;15(5):e0232937. doi: 10.1371/journal.pone.0232937 (PMC7252592; doi:10.1371/journal.pone.0232937)
Supplement: S2 Appendix — (DOCX) [file pone.0232937.s002.docx]

**Appendix 2. Revised guide for semi-structured interviews**

1. Does your farm produce USDA-certified organic dairy products or is milk production conventional?

2. How did you get started in dairy farming?

1. What’s been the biggest change you’ve experienced in the past several years in regard to operating your farm?
2. How many lactating dairy cows are currently on your farm (including all sites)?
3. How many years since you started working with cattle?
4. Do you plan on continuing to work with cattle for the foreseeable future?

3. What health problems in your cattle are you most concerned about?

1. How do you manage those problems? [Prompt: Do you use vaccines, diagnostics, nutritional management, environmental control, etc.?]
2. How do you manage disease in calves, specifically?
3. [Prompt: How do manage disease in bull calves, specifically? Where do you send you bull calves?]
4. Were there any things you’ve considered doing to reduce disease risk but were unable to actually do?
5. Who makes decisions about health problems on the farm?

4. What is a typical interaction with your veterinarian like?

1. What do you typically discuss with your veterinarian?
2. [Prompts: Do you ever contact your veterinarian with questions? What are your questions?]
3. When would you consult with your veterinarian about antibiotic use?
4. [Prompts: Do they provide valuable advice? Do you ever consult with your veterinarian prior to using an antibiotic?]

5. What do you do if a cow gets mastitis?

1. How do you decide on whether to treat the cow with antibiotics?
2. How do you decide which specific antibiotic to use for treatment?
3. Where do you get information about how to make these decisions?
4. [Prompts (for a – c): From personal experience? Do you follow a protocol? Do you utilize diagnostic testing? Do you consult with your veterinarian? Do you follow treatment guidelines issued by drug manufacturers?]
5. How do you keep track of treated animals?
6. [Prompt: Herd management software, individual animal identification,…]
7. What do you understand the term “antibiotic” to mean?

6. Do you ever use diagnostic testing before treating cattle with antibiotics?

1. What tests do you use and when? [Or: Why don't you use diagnostic testing?]
2. Has your veterinarian ever recommended diagnostic testing?
3. Are there differences in you approach to heifers versus cows?

7. Do you have ideas about the use of antibiotics in dairy farming that you think consumers or others should hear?

1. Have consumers’ opinions and preferences affected you?
2. In general (given consumer concerns), do you think dairy farmers could realistically further reduce their antibiotic use?
3. [Probes: Given consumers’ perceptions, do you think any dairy farmers overuse antibiotics?]
4. Why might a farmer not be able to reduce antibiotic use even if he/she would like to??
5. [What resources or information would this farmer need to reduce their antibiotic use?]
6. Is antibiotic use/antibiotic resistance something you talk about with others you know in the business?
7. What would you want to know about other farmer’s use of antibiotic use?

8. What does antibiotic resistance mean to you?

1. Are you concerned about anything related to antibiotic resistance in your farm?
2. Do you know anyone who has been affected by this (human health or animal health)?
3. Where do you get your information about antibiotic resistance?
4. What does the “judicious use of antibiotics” mean to you?

9. What do you think of the Veterinary Feed Directive (VFD)?

(“A VFD drug is intended for use in animal feeds, and such use of the VFD drug is permitted only under the professional supervision of a licensed veterinarian.” Fda.gov

The VFD went to effect on January 1, 2017.)

1. Has it affected operations on your farm?
2. How did you find out about the VFD and/or other rules and regulations (cooperative-level or government-level)?
3. Are you aware of antibiotics that are considered medically important?
4. (These can be found in FDA guidance document #152 Appendix A.)

10. What do you think the future of dairy farming looks like?

1. Do you think that antibiotic use in dairy farms is changing, or will change, in future? How? Why?
2. Is there anything else you’d like to talk about?
