# Supplementary material for: New York State dairy farmers’ perceptions of antibiotic use and resistance: A qualitative interview study
Source: PLoS One. 2020 May 27;15(5):e0232937. doi: 10.1371/journal.pone.0232937 (PMC7252592; doi:10.1371/journal.pone.0232937)
Supplement: S3 Appendix — (DOCX) [file pone.0232937.s003.docx]

**Appendix 3. Codes for Interviews with Dairy Farmers**

Demographic Codes

1. Farm type

- Conventional
- Organic
- Transitioning to organic

1. Gender

- Male
- Female

1. Role on farm

- Owner
- Manager

1. Farm size

- Total number of lactating cows
- Less than 200
- 200 -699
- 700 or more
- Total number of cows
- Other related repsonse

1. Years of experience with dairy cattle

- Raised on a farm
- Worked on a farm since childhood
- Actual number of years working with cattle
- Not raised on a dairy farm
- Other related response

1. Plans to stay in dairy business

- Yes, for forseeable future
- Uncertain
- No, retiring
- No, leaving for other
- Not discussed

1. College education of the interviewee

- Yes
- No
- Not discussed

1. Regularity of vet visits

- Regular vet visits (at least once per month)
- Irregular vet visits (less than once per month) or only when there is health issue
- Unclear/not discussed

1. Role of antibiotic-use protocols

- Has a protocol
- Doesn’t have a protocol
- Has a protocol for calves only
- Organic farm
- Unclear/not discussed

Information Sources

1. Sources of information about antibiotic use

- Veterinarian
- Dairy industry or agricultural publications
- Pharmaceutical advertisements
- Pharmaceutical sales representatives
- Other dairy farm owners or managers
- Nutritionist
- Personal experience
- Other
- Organic farm
- Unclear/not discussed

1. Sources of information about antibiotic resistance

- Media (television, Internet, newspaper, radio, etc)
- Veterinarian
- Dairy industry or agricultural publications
- I don’t receive information on antibiotic use
- Personal experience
- Other
- Unclear/not discussed

1. Sources of information about the VFD and other regulations

- Veterinarian
- Dairy industry or agricultural publications
- Milk inspector
- Milk cooperative
- Other
- Doesn’t remember
- Unfamiliar with VFD
- Unclear/not discussed

Codes Specific to Organic Farmers

1. Primary reason(s) for becoming an organic farmer (or currently working on transitioning to organic for one interviewee)

- Financial benefits
- Belief in organic principles
- Both of financial benefits and belief in organic principles
- Other

1. Satisfaction and perceived effectiveness of treatments or resources available to organic farmers
2. Role of veterinarian in diagnostics and treatment of disease on organic farms
3. Frequency of animals needing antibiotics and what happens when this occurs (if discussed by organic farm owners or managers)

Codes for both Conventional and Organic Farmers

1. Organic and conventional farmers' views of each other and/or larger or smaller farms
2. What the word antibiotic means to interviewees
3. What antibiotic resistance means to interviewees
4. What judicious antibiotic use means to interviewees
5. Concerns about antibiotic use and antibiotic resistance
6. Perceived benefits of reducing antibiotic use and/or downsides of overuse
7. Perceived downsides of reducing antibiotic use
8. Conventional farmers’ perceptions of their own antibiotic use
9. Perceptions of other farmers’ antibiotic use
10. Beliefs about own and/or other farmers’ ability to reduce antibiotic use
11. Barriers to reducing antibiotic use and/or improving animal health and/or “changes” farmers would like to make on their farms
12. Views on consumers
13. Opinion of and experience with the Veterinary Feed Directive, other regulations, and/or dairy industry requirements
14. Opinion of veterinary involvement on dairy farm
15. Factors that influence antibiotic use decisions
